# Double helical π-aggregate nanoarchitectonics for amplified circularly polarized luminescence

**DOI:** 10.1038/s41467-022-29396-0

**Published:** 2022-03-31

**Authors:** Yuan Wang, Dian Niu, Guanghui Ouyang, Minghua Liu

**Affiliations:** 1 Beijing National Laboratory of Molecular Sciences and CAS Key Laboratory of Colloid, Interface and Thermodynamics, Institute of Chemistry, Chinese Academy of Sciences, North First Street 2, Zhongguancun, Beijing, 100190 China; 2 University of Chinese Academy of Sciences, No.19(A) Yuquan Road, Beijing, 100049 China

**Keywords:** Self-assembly, Optical materials, Self-assembly

## Abstract

The canonical double helical π-stacked array of base pairs within DNA interior has inspired the interest in supramolecular double helical architectures with advanced electronic, magnetic and optical functions. Here, we report a selective-recognized and chirality-matched co-assembly strategy for the fabrication of fluorescent π-amino acids into double helical π-aggregates, which show exceptional strong circularly polarized luminescence (CPL). The single crystal structure of the optimal combination of co-assemblies shows that the double-stranded helical organization of these π-amino acids is cooperatively assisted by both CH-π and hydrogen-bond arrays with chirality match. The well-defined spatial arrangement of the π-chromophores could effectively suppress the non-radiative decay pathways and facilitate chiral exciton couplings, leading to superior CPL with a strong figure of merit (*g*_lum_ = 0.14 and QY = 0.76). Our findings might open a new door for developing DNA-inspired chiroptical materials with prominent properties by enantioselective co-assembly initiated double helical π-aggregation.

## Abstract (web summary)

Synthesized or self-assembled helical architectures advance the development of chiral functional materials. Here the authors report a selective-recognized and chirality-matched co-assembly strategy for the fabrication of fluorescent π-amino acids into double helical π-aggregates with exceptional strong circularly polarized luminescence

## Introduction

Synthesized or self-assembled helical architectures not only provide artificial mimics for naturally occurring exquisite helices, such as double helix DNA and triple helix collagen^1^, but also advance the development of chiral functional materials by helical organization of varied building blocks including small organic molecules^2–7^, nanoparticles^8,9^, polymers^10–13^, ions^14^, and bio-macromolecules^15^. Among these intriguing hierarchically chiral structures, helical self-assembled π-architectures encoded with both chirality and luminescence characteristics might emit inequivalent left-handed or right-handed circularly polarized luminescence (CPL), which has attracted increasing attentions among chemistry and material science communities, because of its promising application prospects in the fields of next-generation displays and cutting-edge photonic technologies^16–24^. An ideal CPL material requires both a large luminescent dissymmetry factor (*g*_lum_) and a high quantum yield (QY) or figure of merit (F = *g*_lum_ × QY)^25–27^. However, the improvement of these two factors is usually at the expense of each other in supramolecular assemblies, and effective strategies to overcome the apparent trade-off between *g*_lum_ and QY are still extremely limited. Helical arrangement of π-chromophores has proven to be a promising approach to improve *g*_lum_ values based on previous theoretical investigations and recent experimental advances^28,29^. However, most of these helical π-aggregates are organized in a single helix conformation and only show moderate *g*_lum_ values. Nature has adopted a more sagacious strategy to stabilize and amplify the helical sense of π-aggregates. For example, the double helical π-stacking of the complementary DNA nucleotides with the same absolute configurations is exquisitely fixed in the presence of multiple H-bonds, which has enlightened H-bonds-assisted co-assembly strategy of hetero π-systems to improve the performance of organic π-materials^30–38^. More interesting one is the enantioselective co-assembly among chiral π-systems with different molecular chirality^39,40^, which might not only further manipulate CPL performance through DNA-like hetero π-stacking but also contribute to understanding the origin of homochirality in nature^41^.

**Fig. 1:**
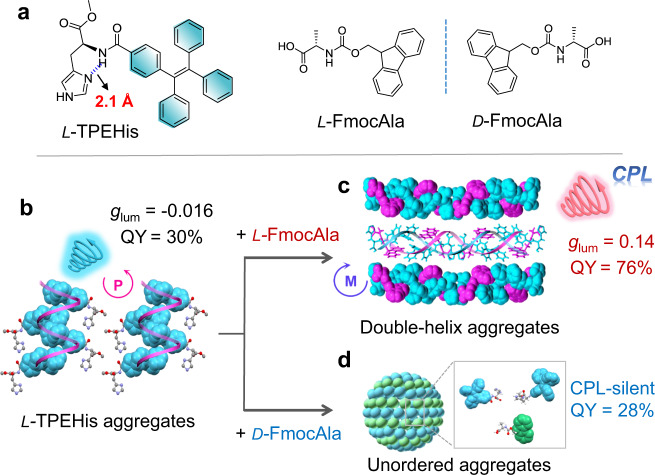
Schematic representation of double helical π-aggregation through enantioselective co-assembly. **a** Molecular structures of histidine amphiphile *L*-TPEHis and Fmoc-protected alanine amphiphile enantiomers, *L*-FmocAla and *D*-FmocAla, respectively. **b** *L*-TPEHis molecules self-assemble into single helix TPE-aggregates. **c** Enantioselective co-assembly of *L*-TPEHis and *L*-FmocAla results in the formation of double helical π-aggregates, showing synchronously amplified *g*_lum_ and FLQY values. **d** Random co-assembly of *L*-TPEHis and *D*-FmocAla leads to unordered aggregates (0-D nanospheres) and quenched CPL emission. *P* and *M* in (**b**, **c**) represent right-handed and left-handed helicity of the helical aggregates, respectively.

Imidazole-mediated H-bonding interactions play important roles in the formation of helical conformations of peptides and histidine proton shuttle functions of a series of enzymes^42^, which have also inspired the development of functional chiral materials based on histidine derivatives. We previously discovered that the imidazole heading groups in a series of chiral π-histidine amphiphiles could form directional H-bonds with amide and carboxylic acid groups, which significantly affected the nanoassemblies and chiroptical properties of single chromophore π-aggregates^43,44^. Inspired by these progress and the H-bonds ruggedized hetero π-stacks of DNA, we conjecture that the imidazole-mediated hydrogen bonds among a chiral π-histidine and a counterpart chiral carboxylic π-system might direct the formation of double helical π-aggregates and accordingly amplify CPL intensities of the co-assemblies. Herein, we report such an enantioselective co-assembled double helical π-aggregates from a binary system composed of chiral tetraphenylethylene-histidine (TPEHis) and fluorenylmethoxycarbonyl-alanine (FmocAla). We found that the cooperative co-assembly of TPEHis and FmocAla with the same molecular chirality (*L*/*L* or *D*/*D*) led to the formation of intertwined TPE/Fmoc π-aggregates with significantly boosted CPL signals compared with single helix TPE-aggregates. While random co-assembly of TPEHis and FmocAla with opposite chirality (*L*/*D* or *D*/*L*) resulted in CPL quenching due to irregular spatial organization of π-luminophores (Fig. 1). Single crystal X-ray crystallography, computational modeling and spectroscopy data suggested that the H-bonds assisted double helical π-stacking of TPE/Fmoc could further reduce the non-radiative decay rate of TPE chromophores and facilitate asymmetric exciton couplings, which contribute to the significant enhancements of both QY and *g*_lum_ values, respectively.

## Results

### Self-assembly of *L*-TPEHis

**Fig. 2:**
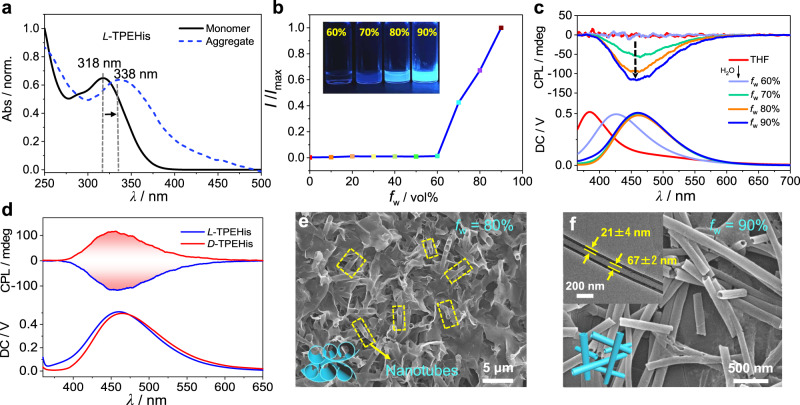
Self-assembly of TPEHis. **a** UV–Vis absorption spectra of monomeric and aggregated *L*-TPEHis. **b** The relative fluorescence intensity (***I***/***I***_max_) of TPEHis versus *f*_w_ (***I*** is the fluorescence intensity of TPEHis under different *f*_w_; ***I***_max_ is the FL intensity at *f*_w_ = 90%; the inserted image is the photograph of TPEHis aggregates with different *f*_w_ values under UV light irradiation, *λ*_ex_ = 365 nm). **c** *f*_w_–dependent CPL spectra of *L*-TPEHis. **d** CPL spectra of *L*-TPEHis and *D*-TPEHis aggregates at *f*_w_ = 90%. **e**, **f** SEM images of *L*-TPEHis assemblies at different *f*_w_ values, the inserted image in (**f**) is the TEM image of an isolated TPEHis nanotube. Unless otherwise noted, [TPEHis] = 10 mM for assemblies. For fluorescence and CPL measurements, *λ*_ex_ is 320 nm.

In the initial molecular design of π-histidine we used tetraphenylethylene (TPE) as the π-chromophore due to its well-known aggregation-induced emission property^45^, which should alleviate the fluorescence quenching phenomenon of helical π-aggregates. Global energy-optimized structure and 2D NMR spectra of TPE-histidine amphiphile support a folded conformation with an intramolecular hydrogen bond between amide and imidazole groups (Fig. 1a, Supplementary Figs. 1–2). UV–Vis absorption spectrum of the monomeric TPEHis in dilute THF exhibited an absorption maximum at 318 nm (Fig. 2a), which was mainly originated from the HOMO-LUMO transition according to TD-DFT computation (Supplementary Figs. 3–4 and Supplementary Table 1)^46^. As expected, the fluorescent activity of TPEHis in dilute solution is prohibited owing to the intramolecular rotation caused non-irradiative relaxations. Adding anti-solvent water into the THF solution of TPEHis leads to the formation of aggregates and significantly increases the fluorescence brightness with water fractions (*f*_w_) over 70% (Fig. 2b and Supplementary Fig. 5). The morphologies of the luminescent *L*-TPEHis aggregates were carefully analyzed. Scanning electron microscopy (SEM) images showed clear dimensional transition from two-dimensional (2-D) curved nanosheets (*f*_w_ 70%) to partially rolled-up nanosheets (*f*_w_ 80%) and 1-D nanotubes (*f*_w_ 90%) upon increasing *f*_w_ values (Supplementary Figs. 6, 2e–f). Transmission electron microscopy (TEM) image reveals that the straight nanotubes have an averaged inner diameter of about 67 ± 2 nm and a wall thickness of about 21 ± 4 nm (Fig. 2f, inserted figure and Supplementary Fig. 7). The formation of helical TPE-aggregates at higher *f*_w_ was probed using both circular dichroism (CD) and CPL spectroscopies. The monomeric TPEHis in dilute THF solution exhibits CD and CPL silence due to impotent chirality transfer from the stereogenic center of histidine to TPE chromophore (Fig. 2c and Supplementary Fig. 8a, red lines). Upon aggregation, the *f*_w_–dependent CPL signals of *L*-TPEHis gradually increased with a |*g*_lum_| value up to 0.016 at 450 nm (Fig. 2c), suggesting the directed formation of helicity preferred TPE-aggregates. This conclusion was further supported by the mirror-imaged Cotton effects and CPL signals of *L*-TPEHis and *D*-TPEHis aggregates (Fig. 2d, Supplementary Fig. 8b).

### Enantioselective co-assembly of *L*-TPEHis with FmocAla

**Fig. 3:**
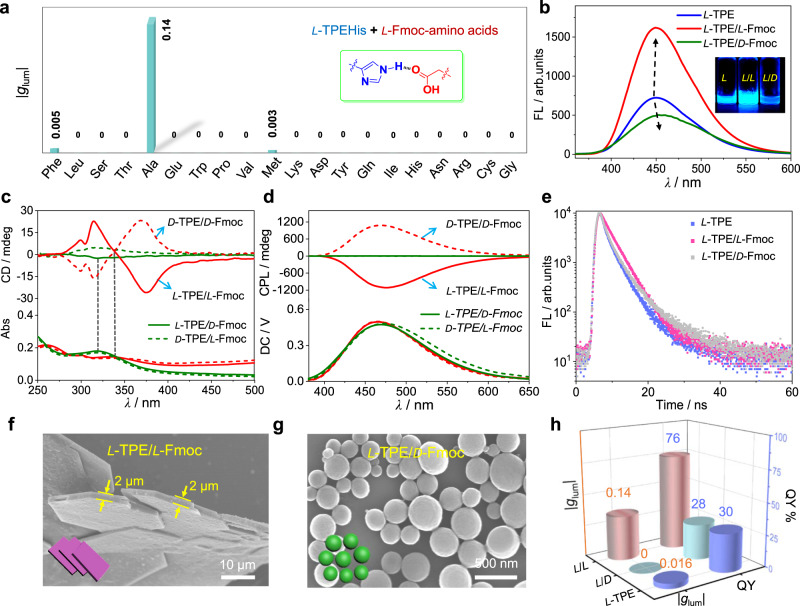
Enantioselective co-assembly of *L*-TPEHis with Fmoc amino acids. **a** Co-assembly of *L*-TPEHis with 20 Fmoc-protected essential amino acids led to selective recognition of *L*-Fmoc-alanine by CPL. **b** Fluorescence spectra of *L*-TPE, *L*-TPE/*L*-Fmoc, and *L*-TPE/*D*-Fmoc assemblies, the inserted figure is sample vials under UV light irradiation. **c** CD and **d** CPL spectra of TPE/Fmoc co-assemblies. *L*/*L*, *L*/*D*, *D*/*D*, *D*/*L* represent different chirality combinations of the two enantiomeric amino acids. **e** The emission decay curves of *L*-TPE/*L*-Fmoc, *L*-TPE/*D*-Fmoc, and *L*-TPE assemblies. **f**, **g** SEM images of *L*-TPE/*L*-Fmoc and *L*-TPE/*D*-Fmoc assemblies, respectively, the inserted figures at the bottom left corners are cartoon representations of the nanostructures. **h** Comparison of *g*_lum_ and QY values of *L*-TPE/*L*-Fmoc, *L*-TPE/*D*-Fmoc, and *L*-TPE assemblies. All the spectroscopic measurements are conducted by transferring the suspensions into cuvettes. Possible contributions from linear dichroism (LD) and linear birefringence (LB) caused by macroscopic anisotropy during the CD and CPL measurements were eliminated (Supplementary Fig. 16). TPEHis and FmocAla are abbreviated as TPE and Fmoc for clarity in Figs. 3–6.

Further enantioselective co-assembly of *L*-TPEHis with a library of Fmoc-amino acid enantiomers were systematically investigated by spectroscopic and morphological analysis to examine our hypothesis, i.e., hetero double helical π-stacking to regulate CPL performance. Our approach to select Fmoc amino acids as the complementary π-systems has the following advantages. First, enantiomeric Fmoc-protected amino acids covering the 20 essential amino acids are commercially available and make it easy to screen a suitable partner for TPEHis with satisfied structural and chirality match (Supplementary Fig. 9). Second, the emission band of Fmoc chromophore is not overlapped with TPE chromophore (Supplementary Fig. 13), which allows us to identify the emission contribution from TPEHis stacks in the binary systems. For experiments, equal mole amounts of *L*-TPEHis and enantiomerically pure Fmoc-amino acids (*L*-enantiomers) were dispersed and dissolved in THF (40 μL), then an excess amount of water (360 μL) was added into the above solution. We could observe the formation of white suspension immediately. Remarkably, the side chains of Fmoc-amino acids have significant influences to the co-assembly and chiroptical outputs. Among the 20 Fmoc-protected essential amino acids (Fig. 3a, Supplementary Figs. 9–11), only the TPEHis/FmocAla co-assemblies exhibited strong CPL emission, thus provides a selective recognition method for FmocAla among these amino acid derivatives. We observed that the fluorescence intensity of *L*-TPEHis/*L*-FmocAla co-assemblies is enhanced obviously compared with *L*-TPEHis assemblies, while *L*-TPEHis/*D*-FmocAla aggregates even showed slightly decreased brightness (Fig. 3b), suggesting an enantioselective discrimination for FmocAla enantiomers. This conclusion was also evidenced by CPL and CD measurements. *L*-TPEHis/*L*-FmocAla and *D*-TPEAla/*D*-FmocAla co-assemblies exhibited outstanding CPL emission, giving an ellipticity value near 1100 mdeg and a |g_lum_| value of 0.14 at 450 nm, which are among the largest values from small organic molecules and their supramolecular assemblies to the best of our knowledge (Fig. 3d). In sharp contrast, the *L*/*D* or *D*/*L* combinations did not show any CPL signals, indicating the formation of unordered aggregates. CD spectra also gave similar differential Cotton effects (Fig. 3c). These results clearly support an enantioselection mechanism during the formation of helical aggregates. We further investigate the co-assembly of *L*-TPEHis and *L*-FmocAla/*D*-FmocAla mixture (in different ratios). The CPL spectra results indicate that *L*-TPEHis trends to co-assemble with the excess enantiomer in the *L*-FmocAla/*D*-FmocAla mixture, while the racemic FmocAla compounds lead to silent CPL signals (Supplementary Fig. 15).

Compared with the monosignate Cotton effect of *L*-TPEHis assemblies at absorption maximum wavelength, CD spectra of *L*/*L* or *D*/*D* co-assemblies exhibited obvious bisignate Cotton effects with a crossover at 338 nm, exactly corresponding to the absorption maximum of TPEHis aggregate (338 nm, Supplementary Figs. 12, 3c). Meanwhile, the absorption dissymmetry factor (*g*_abs_) of *L*-TPEHis/*L*-FmocAla co-assemblies was amplified compared to *L*-TPEHis assemblies (Supplementary Fig. 14a). These results unambiguously indicate strong chiral exciton couplings within co-assembled π-aggregates compared with the individual TPE-stacks. The different π-stacking modes also affect their nanostructures upon further assembly as evidenced from the morphological analysis. SEM micrographs revealed that the nanotube structures of pure TPEHis were changed into thick microplates with crystal-like faces (Fig. 3f, Supplementary Fig. 17a–b), implying highly ordered molecular stacking within *L*/*L* or *D*/*D* co-assemblies, while uneven-sized spherical nanostructures were observed in *L*/*D* or *D*/*L* systems (Fig. 3g, Supplementary Fig. 17c). The compact stacking of TPE chromophores usually promotes AIE process and accordingly improves light-emitting efficiency. We found that the photoluminescent QL of *L*-TPEHis/*L*-FmocAla co-assemblies is significantly increased to 76% compared to the *L*-TPEHis assemblies (30%), while *L*-TPEHis/*D*-FmocAla gave a slightly reduced value (28%), as shown in Fig. 3h, which is in accordance with our above-mentioned conclusion that more compact π-stacks exist in *L*/*L* or *D*/*D* co-assemblies. Time-resolved fluorescence spectra and calculated photophysical parameters show that the non-irradiative decay rate (*K*_nr_) in *L*/*L* or *D*/*D* co-assemblies is greatly reduced, giving a small *K*_nr_ value of 0.81 × 10^8 ^S^−1^ (Fig. 3e, Supplementary Fig. 18, for detailed photophysical data, see Supplementary Table 2 in the supplementary information).

### Single crystal structures

**Fig. 4:**
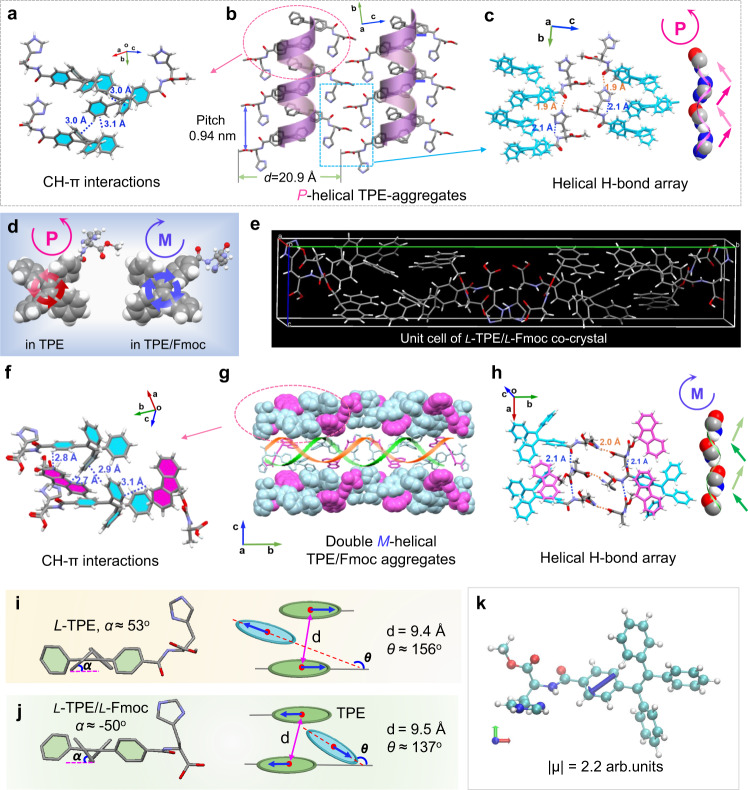
Single-crystal structures revealing the formation of single and double helical π-aggregates. **a**–**c** The single-crystal structure of *L*-TPE and its structural features. **d** The rotational configurations of TPE chromophore in *L*-TPE (left) and *L*-TPE/*L*-Fmoc (right) crystals. **e** The unit cell of *L*-TPE/*L*-Fmoc co-crystal. **f**–**h** The co-crystal structure of *L*-TPE/*L*-Fmoc and its structural features, **g** consists of three parallel stacked unit cells and the middle one was covered with a double-helix cartoon figure. **i**, **j** The average dihedral angel (α) of benzene ring/ethene plane (left figures) and the geometry relations of three nearest neighboring TPE chromophores (right figures) in *L*-TPE (upper) and *L*-TPE/*L*-Fmoc (bottom) crystals. The blue arrows indicate the direction of TPE transition moment. **k** Illustration of S_0_-S_1_ electric transition moment (μ) of TPEHis calculated by TD-DFT at B3LYP 6-311G** level and visualized by VMD program^51^, the *z* axis points outside the plane.

To get more insights on the π-stacking and self-assembly structures, single-crystal X-ray crystallography, Fourier-transform infrared spectroscopy (FT-IR), and X-ray diffraction (XRD) technologies were applied. Single crystals are usually one of the strong evidence to support the formation of helical π-aggregates. Fortunately, we successfully obtained the single crystal of *L*-TPEHis and the co-crystal of *L*-TPEHis/*L*-FmocAla mixture and analysed their crystal structures by X-ray crystallography (Supplementary Tables 3–4). The packing diagram of *L*-TPEHis single crystal shows a monoclinic structure with a *P*2_1_ feature of the chiral space group. Figure 4b shows a well-defined right-handed *P*-helical organization of TPE chromophores, in which two nearest neighbor TPEs are antiparallelly arranged and non-covalently bonded with CH-π interactions (Fig. 4a, average C–H to phenyl center distances are about 3.0 Å). The geometry features of the TPE-helix exhibit a helical pitch length of 0.94 nm and a bilayer thickness of about 2.09 nm (Fig. 4b). Interestingly, the intramolecular hydrogen bond between amide H and imidazole N is reserved in the crystal network (Fig. 4c, blue dashed lines, O…H length of 2.1 Å). Moreover, a new intermolecular hydrogen bond between amide O and imidazole H on two neighboring parallel TPE molecules is formed with a O…H length of 1.9 Å (Fig. 4c, yellow dashed lines). These two types of hydrogen bonds expand to a periodic H-bond array along the stacking direction of TPE helix. The space-filling model of the H-bond array unambiguously displayed a similar *P*-helical sense (Fig. 4c), which further confirms the preferred helical organization of TPEHis molecules. The co-crystal of *L*-TPEHis/*L*-FmocAla mixture shows an orthorhombic structure and constitutes a *P*2_1_2_1_2 chiral space group. Two pairs of *L*-TPEHis/*L*-FmocAla form a guanosine-like quartet stack assisted by CH-π interactions (Fig. 4f, H…phenyl center distances 2.7–3.1 Å) and hydrogen bonds. A unit cell consists of two C2 symmetric quartets and exhibits left-handed (*M*) intertwined double helices along the *b* axis (Fig. 4e). Further extension along the *c* axis by parallel stacking of the supramolecular double-helix leads to the formation of a multilamellar structure (Fig. 4g). Interestingly, the folded conformation of TPEHis molecule restrained by intramolecular amide-imidazole hydrogen bond is forced into an extended conformation, which is stabilized by the new intermolecular amide-carbomate hydrogen bonds (Fig. 4h, yellow dashed lines, O…H distance of 2.0 Å). Similarly, the periodic intermolecular amide-carbomate hydrogen bonds array along the *a* axis also exhibits *M*-helicity (Fig. 4h).

Geometry comparison of the single and double helical π-aggregates could provide insights for the origin of improved emission and chiroptical properties in the co-assemblies. First, the rotational conformation of the four benzene rings of TPE in *L*-TPEHis and *L*-TPEHis/*L*-FmocAla are opposite (Fig. 4d). This means that chirality transfer is originated from the opposite helicity of the single and double helix π-aggregates instead of the stereogenic center of histidine, since in both systems histidine possesses the same *L*-type absolute configurations, which should be responsible for the mirror-imaged CD spectra of *L*-TPEHis and *L*-TPEHis/*L*-FmocAla assemblies in Supplementary Fig. 14. However, the observed CPL patterns were similar and showed the same sign, which indicated that the excited state chirality was different from the ground state chirality^47^. Second, the average dihedral angel of the four benzene rings of TPE chromophore with its ethene plane in *L*-TPEHis/*L*-FmocAla co-crystal (−50°, Fig. 4j) is smaller than that in *L*-TPEHis crystal (53°, Fig. 4i), which reflects the different restriction degree of benzene rotation and might be the origin of higher QL in the enantioselective *L*/*L* or *D*/*D* co-assemblies. Third, the center-to-center distance of two nearest parallel TPE chromophores in the TPEHis/FmocAla co-crystal is slightly larger than that in the TPEHis crystal (Fig. 4i–j, right figures and Supplementary Fig. 19), which should lead to a weaker H-aggregation and therefore a stronger emission^48^. Furthermore, the dihedral angle of two nearest antiparallel TPE planes in *L*-TPEHis crystal is about 156° while in *L*-TPEHis/*L*-FmocAla co-crystal this angel is reduced to 137°. TD-DFT computations indicate that the S_0_-S_1_ electronic transition dipole moment of *L*-TPEHis almost locates within the ethene plane of TPE chromophore and points to the imidazole side along the ethylene double-bond direction (Fig. 4k). According to Kasha’s exciton model^49^, the smaller dipole-dipole angel (137°) in an oblique exciton arrangement system should results in a strong exciton coupling compared with the one with 156° angel. This is in agreement with the enhanced QL and observed exciton-split type Cotton effect in the CD spectrum of *L*-TPEHis/*L*-FmocAla co-assemblies (Fig. 3c, h).

### Self-assembly mechanism

**Fig. 5:**
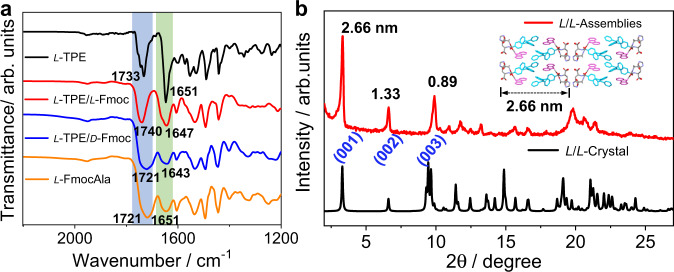
FT-IR and XRD measurements. **a** FT-IR spectra of *L*-TPE, *L*-TPE/*L*-Fmoc, *L*-TPE/*D*-Fmoc, and *L*-FmocAla assemblies. **b** Experimental XRD of *L*-TPE/*L*-Fmoc assemblies (red line) and simulated XRD of *L*-TPE/*L*-Fmoc co-crystal (black line), the inset indicates the bilayer width in the co-crystal.

FT-IR spectra provide further information for understanding the stacking mechanism. The obvious amide I and amide II peaks support the existence of amide hydrogen bonds in both *L*-TPEHis and *L*-TPEHis/*L*-FmocAla assemblies. The peak at 1721 cm^−1^ in *L*-FmocAla assemblies is ascribed to the C=O stretching band of a hydrogen-bonded carboxylic acid dimer (Fig. 5a, orange line). This peak is unchanged in the *L*-TPEHis/*D*-FmocAla assemblies (Fig. 5a, blue line), indirectly supporting a self-sorting aggregation behavior. This conclusion could also be supported by the almost equal PLQY values in *L*-TPEHis and *L*-TPEHis/*D*-FmocAla systems (Fig. 3h). However, the IR peak of the dimeric carboxylic acid is significantly moved to 1740 cm^−1^ in *L*-TPEHis/*L*-FmocAla assemblies (Fig. 5a, red line), demonstrating the disassociation of carboxylic dimer and concomitant formation of new types of carboxylic-imidazole hydrogen bond. Most importantly, the simulated XRD pattern of *L*-TPEHis/*L*-FmocAla single crystal is highly consistent with the experimental XRD peaks of *L*-TPEHis/*L*-FmocAla co-assemblies (Fig. 5b). All these data indicate a similar double helix packing mode in the enantioselective co-assemblies to the co-crystal structure.

**Fig. 6:**
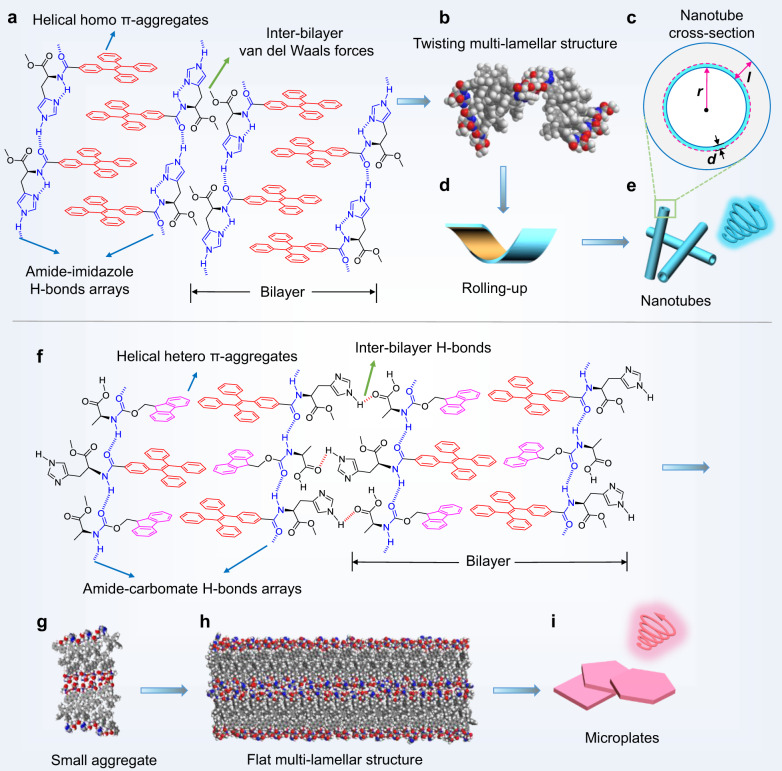
Proposed self-assembly mechanism and computational simulation. **a** Chemical structures of possible packing mode of *L*-TPEHis. **b** PM6-D3H4 method optimized structure of *L*-TPE aggregates showing twisted topology. **c** The cross-section of *L*-TPE nanobute. ***r***, ***l***, and ***d*** represent the radius, wall thickness, and a bilayer width, respectively. **d**–**e** Proposed formation mechanism of nanotubes. **f** Chemical structures of possible packing mode of *L*-TPEHis/*L*-FmocAla co-assemblies. **g** The PM6-D3H4 method and **h** molecular dynamic (MD) simulation^52^ optimized structures of *L*-TPE/*L*-Fmoc co-assemblies show a flat multi-lamellar geometry, which further assembles into microplates (**i**).

To gain further insights into the molecular arrangement within the aggregates, semi-empirical calculations (PM6-D3H4) and molecular dynamic (MD) simulations with Cartesian coordinates extracted from the single crystals were performed. For *L*-TPEHis assemblies, antiparallel arranged TPEHis molecules form a bilayer structure, which is stabilized by π-π interactions and intra- and inter-molecular amide-imidazole hydrogen bond arrays (for chemical structures, see Fig. 6a). The subsequent formation of a multilamellar structure is dominated by van der Waals forces as revealed by the single-crystal structure, which makes the interbilayer orientation sensitive to surrounding conditions. In this case, the multilamellar structure trends to twist and roll up as supported by the PM6-optimized structure, leading to the gradual formation of nanotubes (Fig. 6b, d, e). Based on the statistically analyzed wall thickness of TPEHis nanotubes (*l* = 21 ± 4 nm) and the bilayer width obtained from the single crystal (*d* = 2.1 nm), the number of TPEHis bilayers within the nanotube wall is calculated to be about *l*/*d* = 10 ± 2 (Fig. 6c). For *L*-TPEHis/*L*-FmocAla co-assemblies, hetero TPE/Fmoc chromophores form bilayer structure by π-π interactions and intermolecular amide-carbomate hydrogen bonds (for chemical structures, see Fig. 6f). The directional carboxylic-imidazole hydrogen bond ensures stronger interbilayer interactions and more ordered multibilayers, which lead to the formation of flat multilamellar structure as evidenced from the computational simulation results. As shown in Fig. 6g, the PM6-D3H4 method optimized structure of a small aggregate of TPEHis/FmocAla mixture displays a planar geometry. For larger TPEHis/FmocAla aggregate, a molecular dynamic (MD) simulation was performed to evaluate its equilibrium configuration (Fig. 6h). After MD simulation, a pre-assembled two bilayers containing 120 pairs of TPEHis/FmocAla array also show planar geometry, indicating robust structural stability of the flat multilamellar structure, which is liable for the formation of subsequent microplates with length scale over tens of micrometers (Fig. 6i).

## Discussion

The conformational constraint by double helix requires a complete complementary counterpart for *L*-TPEHis, reminiscent of the A/T and C/G base pairs in DNA double helix. The selectivity of alanine among the 20 Fmoc-protected essential amino-acids indicates that the methyl group on alanine is indispensable to satisfy the double helical conformational requirement. To further investigate the role of Fmoc chromophore, we designed a control experiment by using Boc-protected *L*-alanine and *D*-alanine to co-assemble with *L*-TPEHis. In this case only moderate *g*_lum_ values are obtained (Supplementary Fig. 20), indicating the Fmoc component is also a requirement for the formation of double helix. The calculation results show that the double helical conformation is highly stable in different sizes (Fig. 6g–h), thus further demonstrating the structural complementation of Fmoc-alanine with TPEHis, which should be responsible for the impressive stereo- and enantio-selective co-assembly.

Our results show that double helical π-aggregate can be obtained by enantioselective and chirality-matched co-assembly among hetero π-amino acids. The precise structural and chirality matching of TPEHis and Fmoc amino acids observed in the double helical π-aggregates stresses the advantages of combinatorial screening strategy among different π-systems. Furthermore, inter- and intra-molecular hydrogen bonds play an important role in directing the hierarchical assembly of these helical π-chains, resembling the naturally occurring DNA double helix. Synergistic π-helix and H-bond helix contribute to reinforced helical sense and lead to significantly amplified CPL signals over their individual assemblies. This work provides a bio-inspired approach to effectively improve the collective chiroptical properties of chiral π-systems upon enantioselective co-assembly, which we believe will contribute to deepening our understanding of the origin of homochirality and developing alternative strategy to manipulate chiral supramolecular materials as well as advanced nanoarchitectonics^50^.

## Methods

### Materials

All commercial chemicals were used as received without further purification. Milli-Q water (18.2 MΩ cm) was used in all cases. *L*- and *D*-Histidine methyl ester dihydrochloride, 4-(1,2,2-Triphenylvinyl) benzoic acid were purchased from TCI (purity > 97%). Fluorenylmethoxycarbonyl-acid compounds were purchased from Alfa Aesar (purity 95%). 1-hydroxybenzotriazole (HOBt, purity > 98%), 1-(3-dimethylaminopropyl)-3-ethylcarbodiimide hydrochloride (EDC•HCl, purity 97%) and triethylamine (purity 99%) were purchased from J&K. The synthetic procedures of *L*-TPEHis and *D*-TPEHis were listed in Scheme S1, supplementary information. The products were purified by column chromatography and characterized by ^1^H NMR, ^13^C NMR, and MALDI-FTICR-MS.

### Self-assembly protocol

TPEHis (0.004 mmol) was dissolved in THF (40 μL) upon heating. After cooling to room temperature for 30 min, 360 μL distilled water was added to the above dilute THF solution. A white suspension was obtained immediately and the sample was allowed aging for 24 h before further testing.

### Co-assembly protocol

Equal mole of *L*/*D*-TPEHis (0.004 mmol) and *L*/*D*-Fmoc amino acids (0.004 mmol) were dissolved in THF (40 μL) upon heating. After cooling to room temperature, 360 μL distilled water was added and the mixture were aged for 24 h before next measurements.

### Ultraviolet-visible spectroscopy

UV–Vis spectra were recorded on JASCO UV-550 and Hitachi UV-3900 spectrometers. The samples were prepared for testing on quartz cuvettes with light path of 0.1 mm, and the wavelength scanning range was 250–650 nm.

### Fluorescence spectra

The fluorescence spectra were recorded on a Hitachi F-4600 fluorescence spectrophotometer. The samples were prepared for testing on quartz cuvettes with light path of 0.1 mm, the testing voltage was 400 V, the width of the excitation and emission slits were 4 nm. Fluorescence QLs were measured by absolute method on a FluoroMax + (HORIBA) instrument by using an integrating sphere.

### CD spectra

CD spectra were measured with JASCO 1500 and JASCO 815 spectrophotometers. Samples were prepared for testing in quartz cuvettes with light path of 0.1 mm. The electronic circular dichroism (ECD) and linear dichroism (LD) spectra were recorded, and contributions from LD to CD were very small and could be ignored from our experimental results.

### CPL spectra

CPL spectra were measured with JASCO CPL-200 and JASCO CPL-300 spectrophotometers. The sample was prepared by transferring a droplet of suspensions into a quartz cuvette with light path of 0.1 mm. The linear birefringence (LB) effects and possible artifacts caused by macroscopic anisotropy were generally excluded by flipping and changing the angle of the sample along the direction of incident light propagation.

### NMR and mass spectra

^1^H NMR, ^13^C NMR, COSY and NOESY spectra were recorded on a Bruker ADVANCE III 400 and 600 (^1^H: 400 MHz and 600 MHz, ^13^C: 100 MHz) spectrometer in DMSO-*d*_6_ at 298 K. Mass spectrum data were obtained by using a BIFLEIII matrix-assisted laser desorption/ionization time of fight mass spectrometry (MALDI-FTICR-MS) instrument, the sample was dissolved in THF and volatilized on the substrate for testing.

### Scanning electron microscopy (SEM)

SEM images were recorded on a Hitachi S-4800 FE-SEM instrument with an accelerating voltage of 10 kV, the samples were prepared on single-crystal silicon wafer, dried under vacuum, and coated with a thin layer of Au to increase the contrast.

### Transmission electron microscopy (TEM)

TEM images were obtained on a T20 electron microscope at an accelerating voltage of 110 kV. A small amount of the suspension was diluted with a mixed solvent of THF/H_2_O, dropped on the surface of the copper mesh of the carbon support film, dried in vacuum, and observed under an electron microscope.

### Fourier transform infrared (FT-IR)

FT-IR spectra were recorded on a Bruker TENSOR-27 FT-IR spectrometer, samples were cast on single-crystal silicon wafer and tested after vacuum drying.

### Single crystal X-ray diffraction

SXRD were collected on an XtaLAB Synergy-R diffractometer. The structures were solved by direction methods and refined by a full matrix least squares technique based on F2 using SHELXL 97 program (Sheldrick, 1997). The crystal packing were obtained using the software Mercury 1.4.1. *L*-TPEHis and *L*-TPEHis/*L*-FmocAla crystals suitable for X-ray diffraction were obtained in 1,4-dioxane solution and ethyl acetate solution by a slow evaporation method, respectively.

### X-ray diffraction (XRD) measurements

PXRD analysis was performed on a Rigaku D/Max-2500 X-ray diffractometer with Cu/Kα radiation (*λ* = 1.5406 Å), which was operated at a voltage of 40 kV and a current of 200 mA. Samples were prepared on single-crystal silicon wafer and vacuum dried for PXRD testing.

### Density functional theory (DFT) computation

DFT and time-dependent DFT calculations were performed by Gaussian 09 program at B3LYP 6-311G** level.

### Molecular dynamic (MD) simulation

The molecular dynamic (MD) simulation was implemented with the large-scale atomic/molecular massively parallel simulator (LAMMPS) packages. The cut-off distance for non-bonded interactions was set to 11 Å. The geometric mixing rule were applied to describe the van der Waal (VdW) interaction between the inter-molecules of the system. Energy minimization was conducted using the conjugate gradient algorithm before performing dynamic simulations. MD simulations for all systems were carried out for 15 ns with a time step of 1 fs per integration step under the ensemble conditions of *T* = 298 K. All simulations were visualized using OVITO program. The simulation box sizes were fixed as 25 × 12 × 10 nm^3^. The CVFF force field was employed to describe interatomic interactions between molecules.

### Reporting summary

Further information on research design is available in the Nature Research Reporting Summary linked to this article.

## Supplementary information

Supplementary Information

Reporting Summary

## Data Availability

All relevant data are available from the authors. Supplementary Information is available in the online version of the paper. The X-ray crystallographic coordinates for structures reported in this article have been deposited at the Cambridge Crystallographic Data Centre, under deposition number CCDC: 2129359, 2129360. Source data are provided with this paper.

## Source data

Source Data
