# Supplementary material for: Double helical π-aggregate nanoarchitectonics for amplified circularly polarized luminescence
Source: Nat Commun. 2022 Mar 31;13:1710. doi: 10.1038/s41467-022-29396-0 (PMC8971395; doi:10.1038/s41467-022-29396-0)
Supplement: Supplementary file 1 — Supplementary Information [file 41467_2022_29396_MOESM1_ESM.pdf]

Supplementary Information for

Double Helical π-Aggregate Nanoarchitectonics for Amplified Circularly Polarized Luminescence

Yuan Wang,[1,2,3] Dian Niu,[1,3] Guanghui Ouyang,[1]* and Minghua Liu[1,2]*

[1]Beijing National Laboratory of Molecular Sciences and CAS Key Laboratory of Colloid, Interface and Thermodynamics, Institute of Chemistry, Chinese Academy of Sciences, North First Street 2, Zhongguancun, Beijing 100190, China

[2]University of Chinese Academy of Sciences, No.19(A) Yuquan Road, Beijing 100049, China

[3]These authors contributed equally

*Correspondence to: ouyanggh@iccas.ac.cn, liumh@iccas.ac.cn

Contents

1. Synthetic procedures and characterization..3

2. Density functional theory calculations..6

3. Self-assembly of TPEHis...7

4. Co-assembly of TPEHis with Fmoc amino acids 9

5. Co-assembly of TPEHis with FmocAla...12

6. Single crystal data 18

7. Additional spectra 20

8. References...22

1. Synthetic procedures and characterization

Synthesis of *L*-TPEHis: *L*-(+)-histidine methyl ester dihydrochloride (0.97 g, 4.0 mmol) was dispersed in dry dichloromethane (100 mL) in a 250 mL round flask. Triethylamine (1.01 g, 10.0 mmol) was added and the mixture was stirred at R.T. for 1 h. 4-(1,2,2-triphenylvinyl) benzoic acid (1.25 g, 3.33 mmol), 1-hydroxybenzotriazole (HOBt, 0.58 g, 4.33 mmol) and 1-(3-dimethylaminopropyl)-3-ethylcarbodiimide hydrochloride (EDC•HCl, 0.83 g, 4.33 mmol) were added to the solution and was stirred at R.T. overnight. After the reaction was finished, the reaction mixture was washed with saturated $NaHCO_3$ aqueous solution for three times (3×200 mL). The organic phase was collected and dried over anhydrous Na_2SO_4. After being filtered, the organic solvent was removed using rotary evaporator and the obtained crude product was purified by flash column chromatography. The product was white solid (1.18 g, 2.24 mmol, yield 67.3 %).

The synthetic procedures of *D*-TPEHis enantiomer are the same with that of *L*-TPEHis.

^{1}H NMR (400 MHz, DMSO-d_6, 298K, δ, ppm): 11.91 (s, 1H), 8.81 (d, J = 7.2 Hz, 1H), 7.62-7.56 (m, 3H), 7.14-6.97 (m, 17H), 6.84 (s, 1H), 4.62 (m, 1H), 3.61 (s, 3H), 3.00 (d, J = 6.9 Hz, 2H).

^{13}C NMR (100 MHz, DMSO-d_6, 298K, δ, ppm): 172.61, 166.26, 147.08, 143.31, 143.20, 142.00, 140.22, 135.51, 131.92, 131.11, 131.06, 128.40, 127.37, 127.20, 53.53, 52.33, 31.79.

HR-MALDI-TOF MS: m/z calcd. for $[M+H]^+$ $C_{34}H_{29}N_3O_3$: 528.22817. Found: $[M+H]^+$, 528.22854.

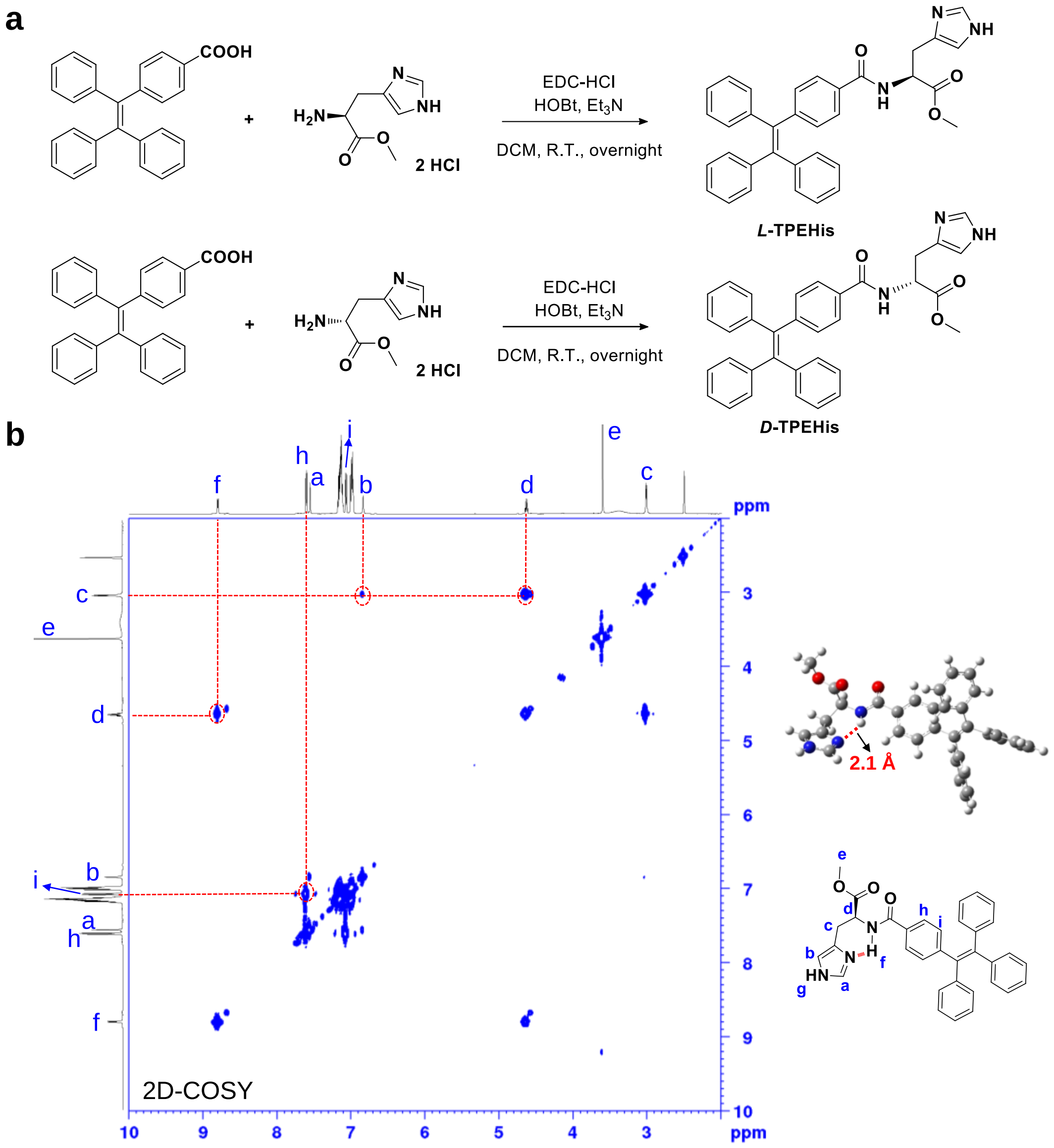

Supplementary Figure 1. a. Synthetic routes to *L*- and *D*-TPEHis. **b**. 2D H-H COSY NMR spectrum of *L*-TPEHis (600 MHz, DMSO-d_6, 298K) and assignment of hydrogen atoms. Upper right figure in **b** is the DFT-optimized geometry of *L*-TPEHis at B3LYP 6-311G** theory level.

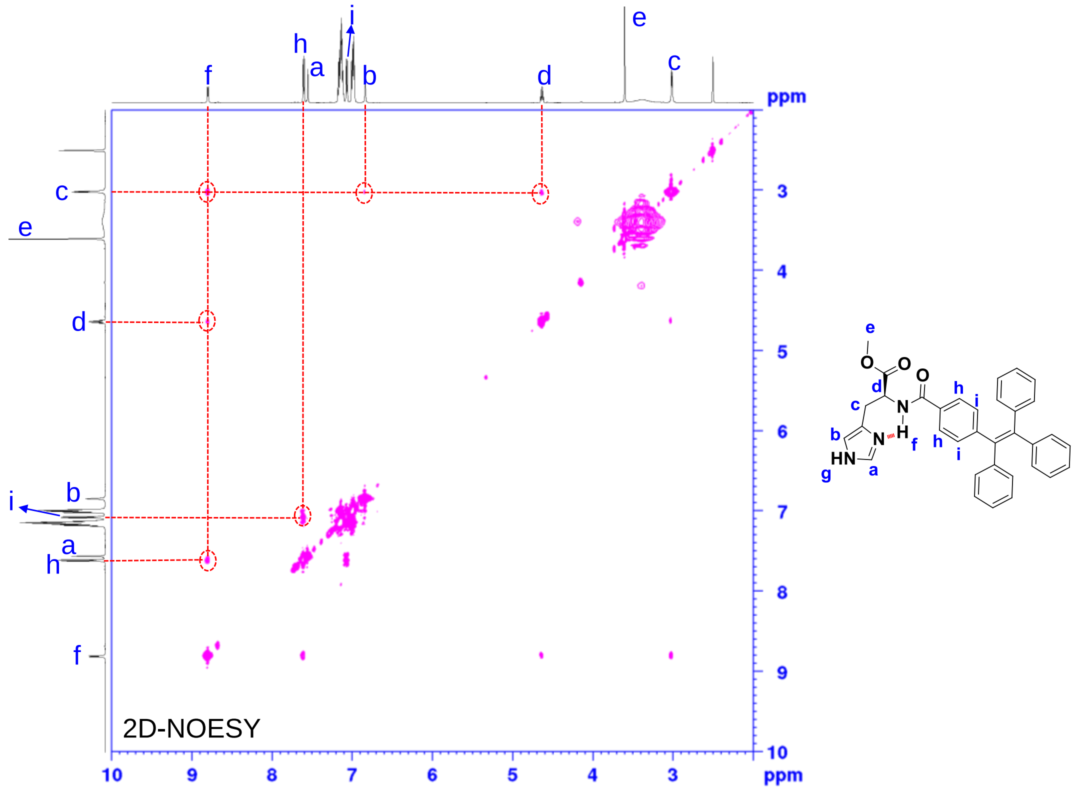

Supplementary Figure 2. 2D H-H NOESY NMR spectrum of *L*-TPEHis (600 MHz, DMSO-d_6, 298K) and assignment of hydrogen atoms.

2. Density functional theory calculations

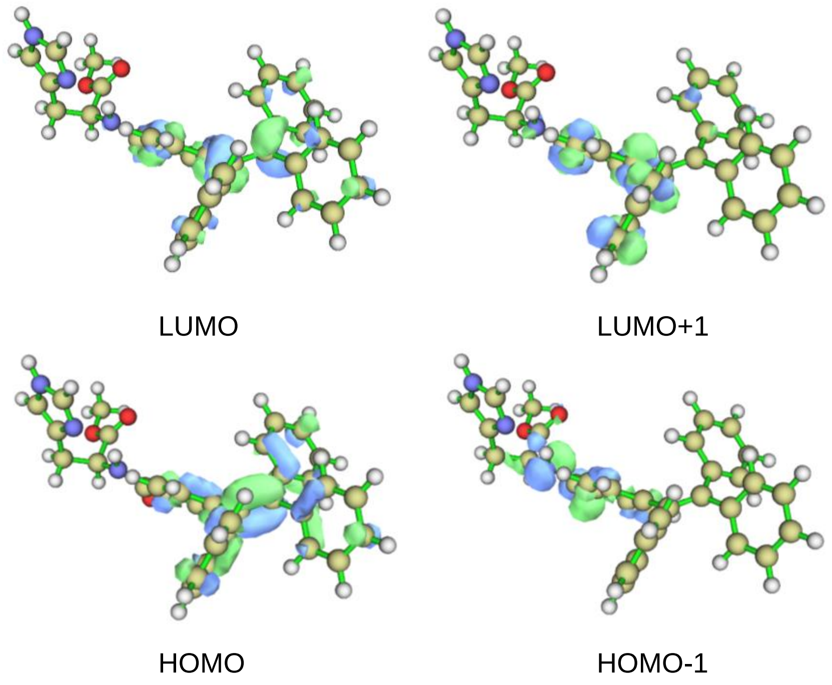

Supplementary Figure 3. Frontier molecular orbitals calculated by DFT at B3LYP 6-311 G** level[1]. The blue and green colors of electron cloud represent hole and electron distributions, respectively. The red, cyan orange, blue and gray balls represent oxygen, carbon, nitrogen, and hydrogen atoms, respectively[2].

Supplementary Table 1. Excited states and molecular orbital (MO) transition contributions[a].

States	MO contribution	Energy	Wavelength	Oscillator strength
S1	HOMO → LUMO (99.1%)	3.58 eV	347 nm	0.436
S2	HOMO → LUMO+1 (94.3%)	4.20 eV	296 nm	0.147
S3	HOMO-1 → LUMO (49.3%)	4.30 eV	288 nm	0.007
S4	HOMO → LUMO+2 (15.8%)	4.33 eV	286 nm	0.014

[a] Calculated by TD-DFT, Gaussian 09, B3LYP 6-311 G** level.

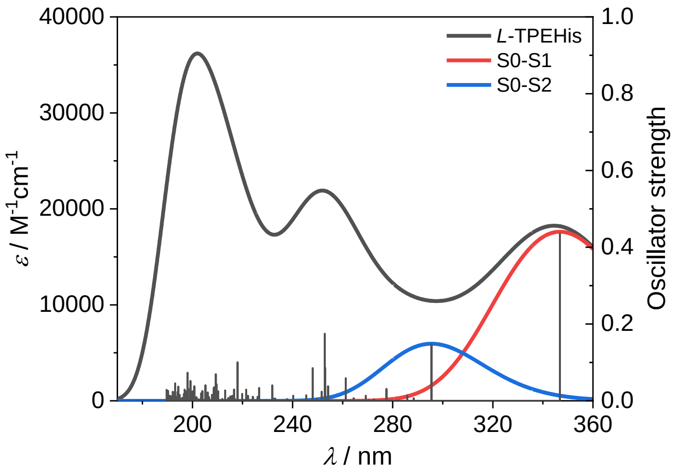

Supplementary Figure 4. Calculated UV-vis spectrum of *L*-TPEHis (black line) and contributions of S_0-S_1 transition (red line), S_0-S_2 transition (blue line). TD-DFT at B3LYP 6-311 G** level. The black vertical lines represent oscillator strength of different electric transitions.

3. Self-assembly of TPEHis

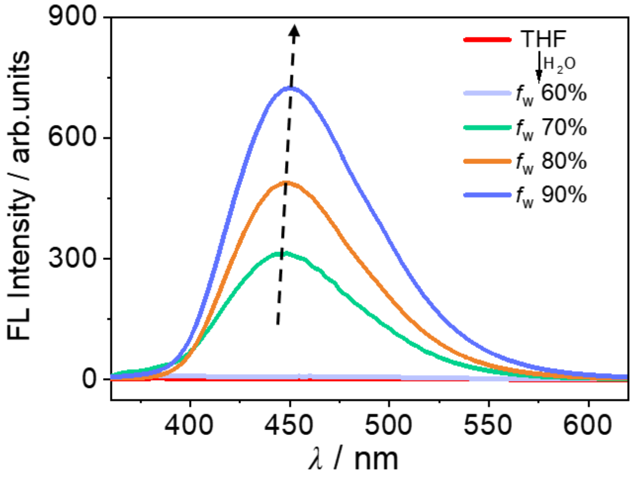

Supplementary Figure 5. f_w-dependent fluorescence spectra of *L*-TPEHis in THF/H_2O mixtures, λ_{ex} = 320 nm. Unless otherwise mentioned, [TPEHis] = 10 mM in all the experiments.

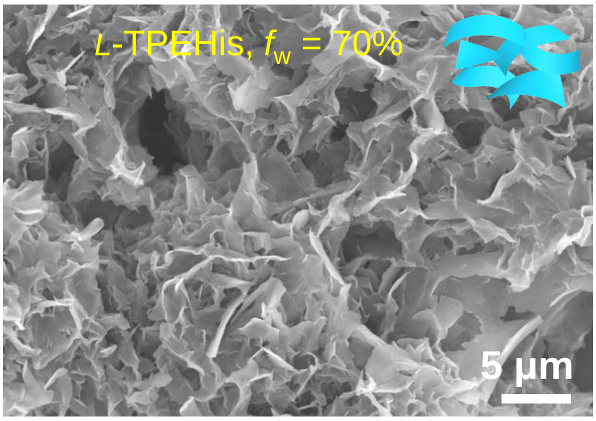

Supplementary Figure 6. SEM image of *L*-TPEHis self-assemblies at f_w = 70%.

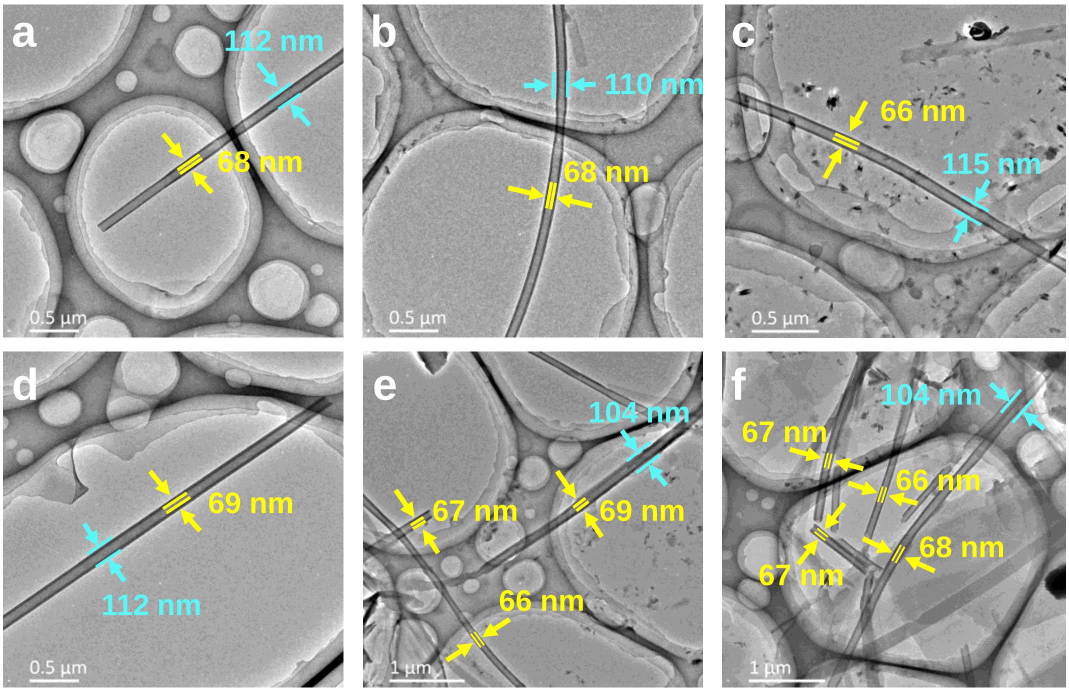

Supplementary Figure 7. TEM images of *L*-TPEHis self-assemblies at f_w = 90%. Average inner diameter of the nanotube is 67±2 nm. Average outer diameter is 110 ±4 nm.

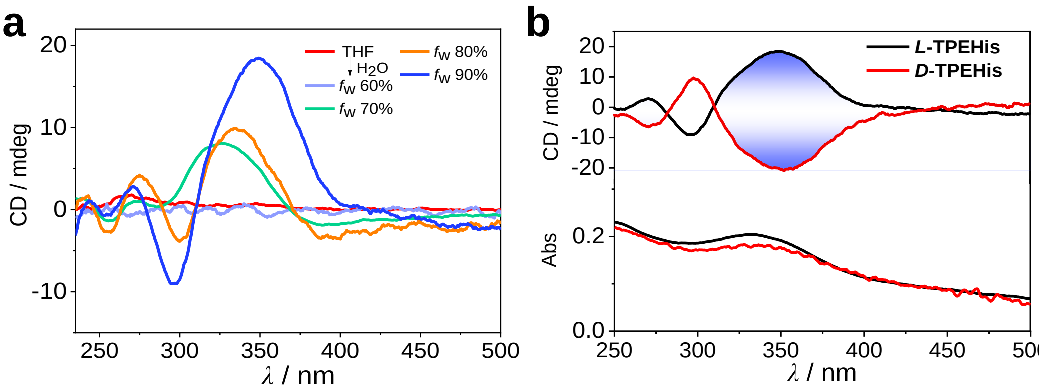

Supplementary Figure 8. a. CD spectra of *L*-TPEHis aggregates upon increasing f_w values. **b**. CD spectra of *L*-TPEHis and *D*-TPEHis aggregates.

4. Co-assembly of TPEHis with Fmoc amino acids

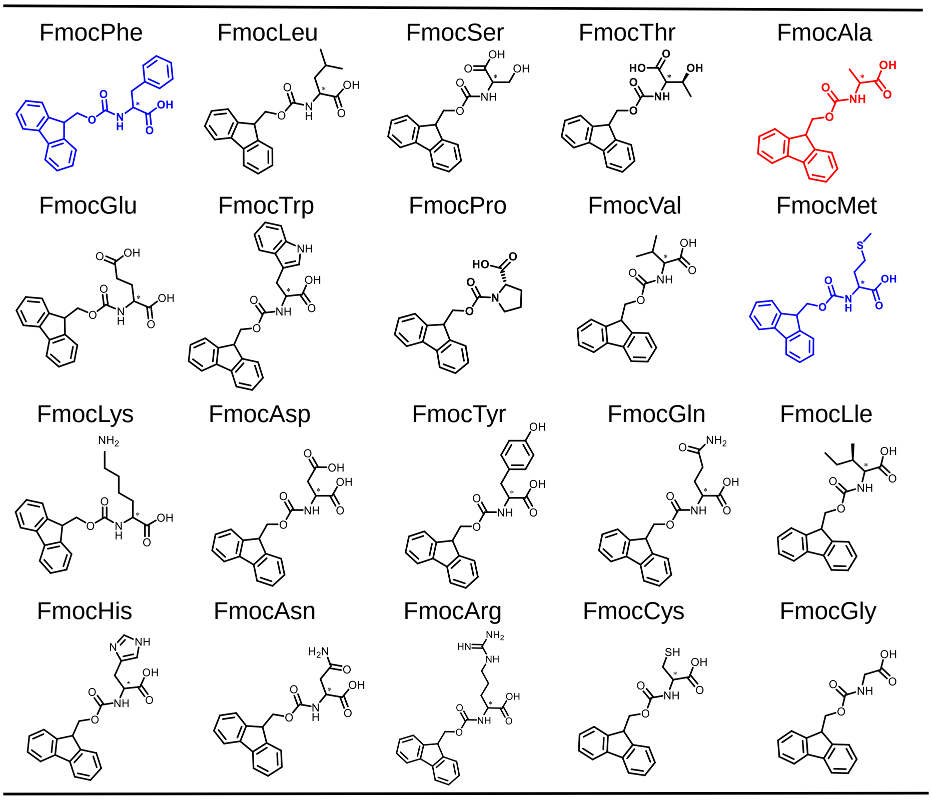

Supplementary Figure 9. Chemical structures of 20 Fmoc-protected essential amino acids investigated here.

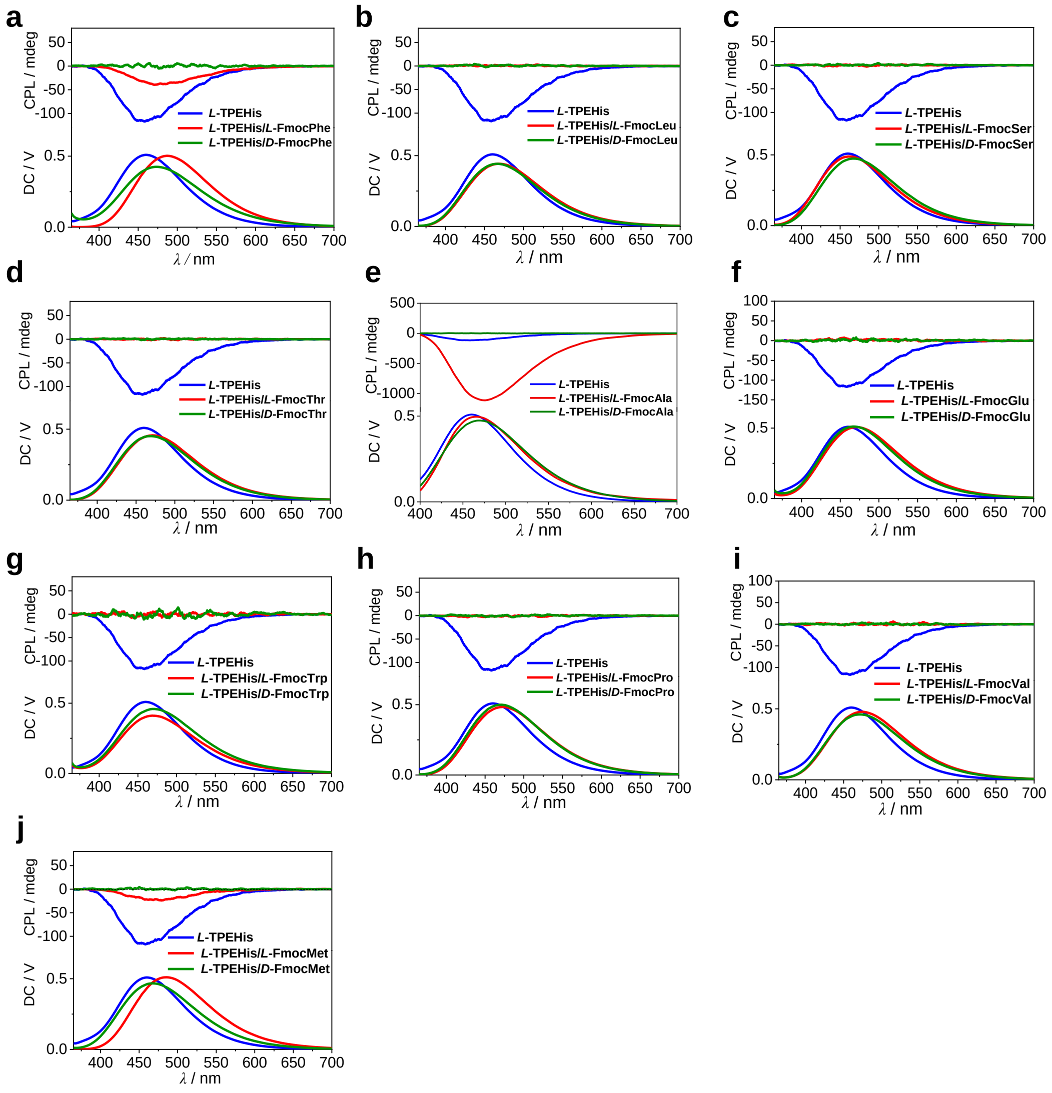

Supplementary Figure 10. CPL spectra of co-assemblies of *L*-TPEHis with *L*-Fmoc amino acids and *D*-Fmoc amino acids. Unless otherwise mentioned, [TPEHis] = [Fmoc amino acids] = 10 mM in THF/H_2O, f_w = 90%, λ_{ex} = 320 nm.

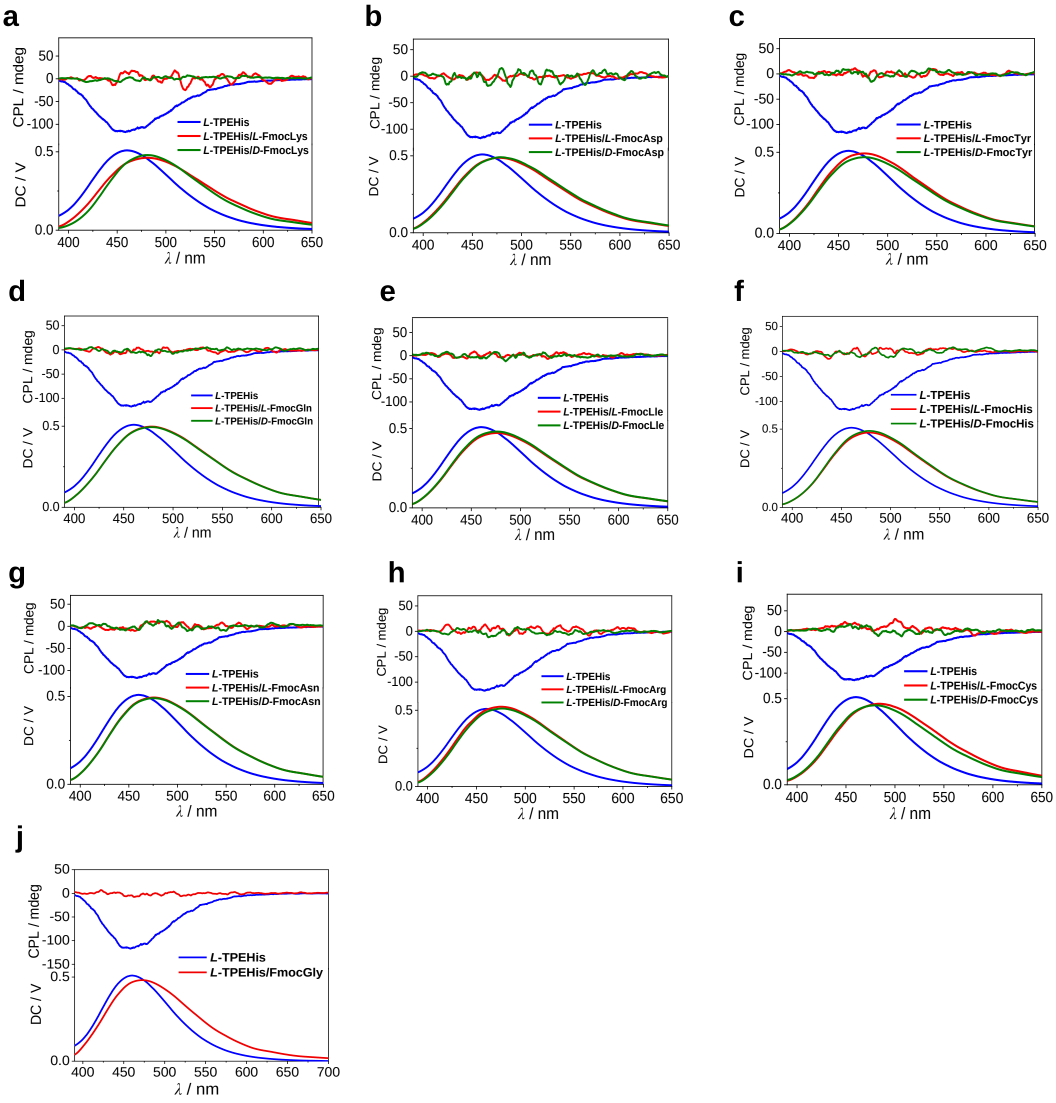

Supplementary Figure 11. CPL spectra of co-assemblies of *L*-TPEHis with *L*-Fmoc amino acids and *D*-Fmoc amino acids.

5. Co-assembly of TPEHis with FmocAla

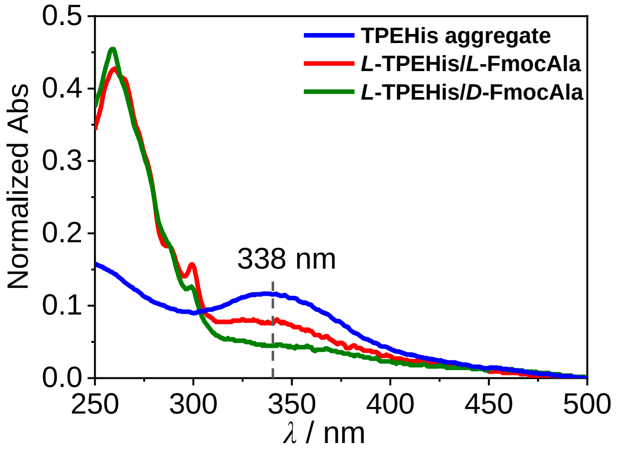

Supplementary Figure 12. UV-Vis spectra of *L*-TPEHis aggregate (blue line), *L*-TPEHis/*L*-FmocAla co-assemblies (red line) and *L*-TPEHis/*D*-FmocAla co-assemblies (green line).

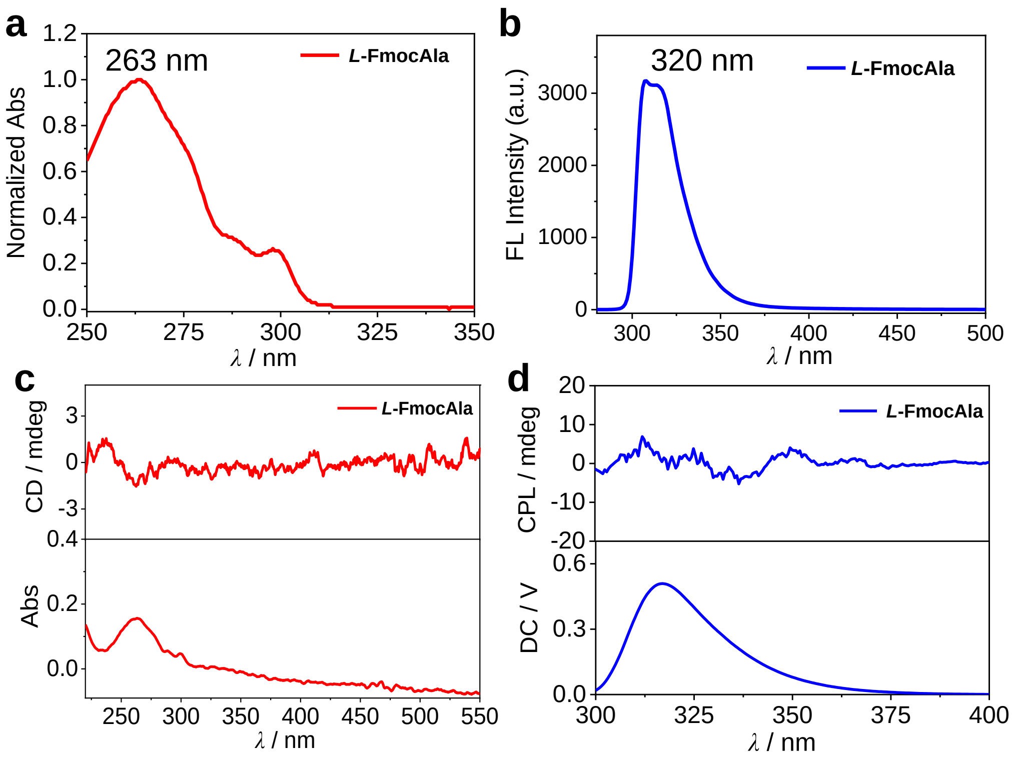

Supplementary Figure 13. Optical and chiroptical spectra of *L*-Fmoc alanine assemblies. **a**. UV-Vis spectrum, **b**. FL spectrum, **c**. CD spectrum, **d**. CPL spectrum. [*L*-Fmoc alanine] = 10 mM, f_w = 90%, λ_{ex} = 260 nm for FL and CPL measurements.

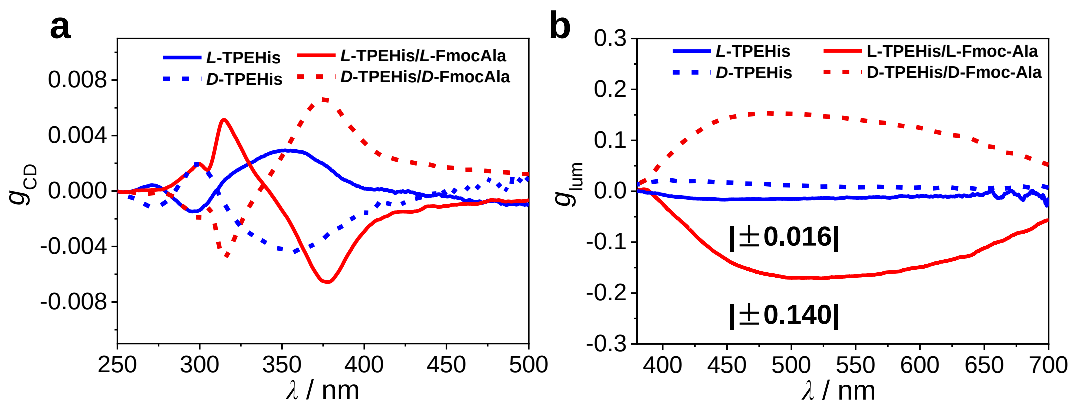

Supplementary Figure 14. a. Absorption dissymmetry factor (g_{abs}), **b**. Luminescent dissymmetry factor (g_{lum}) of assemblies of *L*-TPEHis (solid blue lines), *D*-TPEHis (dashed blue lines), *L*-TPEHis/*L*-FmocAla (solid red lines), *D*-TPEHis/*D*-FmocAla (dashed red lines). λ_{ex} = 320 nm for FL and CPL measurements.

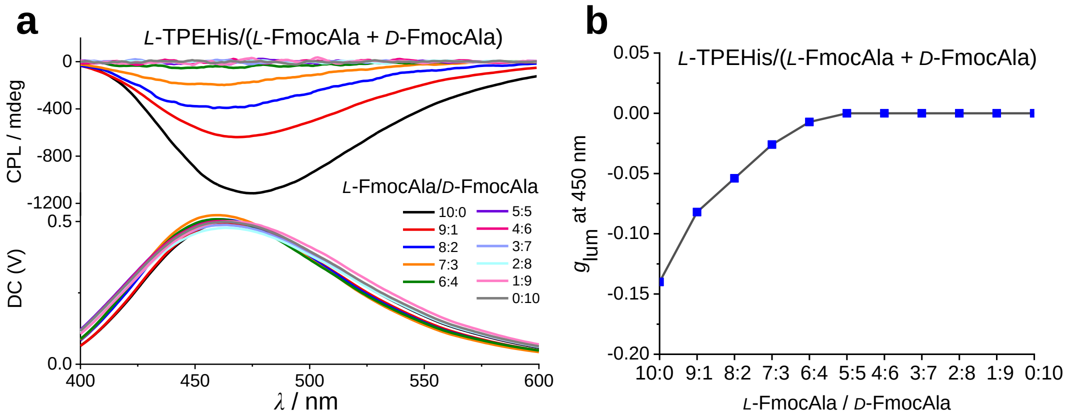

Supplementary Figure 15. a. CPL spectra of the co-assemblies of *L*-TPEHis with *L*-FmocAla/*D*-FmocAla mixture (in different ratios). **b**. g_{lum} values of *L*-TPEHis/(*L*-FmocAla + *D*-FmocAla) co-assemblies against the *L*-FmocAla/*D*-FmocAla ratios. [TPEHis] = [Total FmocAla] = 10 mM in THF/H_2O, f_w = 90%, λ_{ex} = 320 nm.

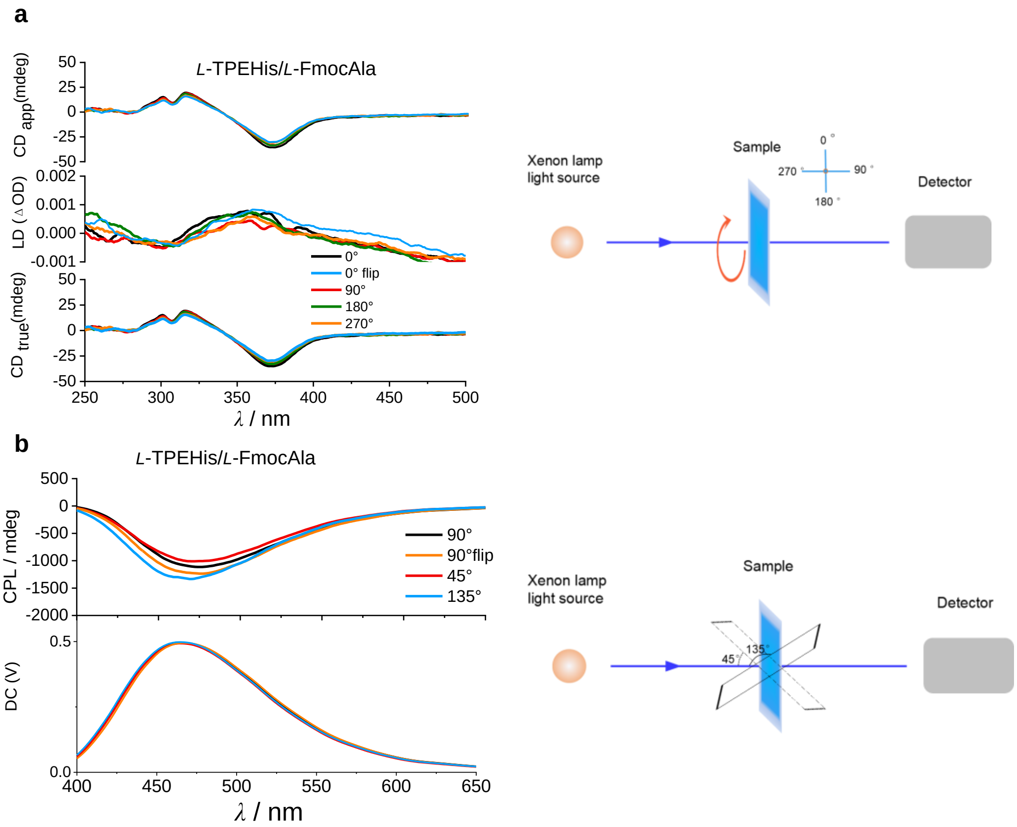

Supplementary Figure 16. a. The contributions of LD to true CD signals (CD_{ture}) according to the approximation equation of $CD_{true} = CD_{app} - 0.02 \times LD$ in the *L*-TPEHis/*L*-FmocAla co-assemblies by flipping and rotating the cuvette each 90º around the incident light propagation direction, CD_{app} represent the apparent CD signals obtained from the CD photospectrometer. **b**. The CPL spectra of *L*-TPEHis/*L*-FmocAla co-assemblies by flipping and changing the angle of the sample along the direction of incident light propagation.

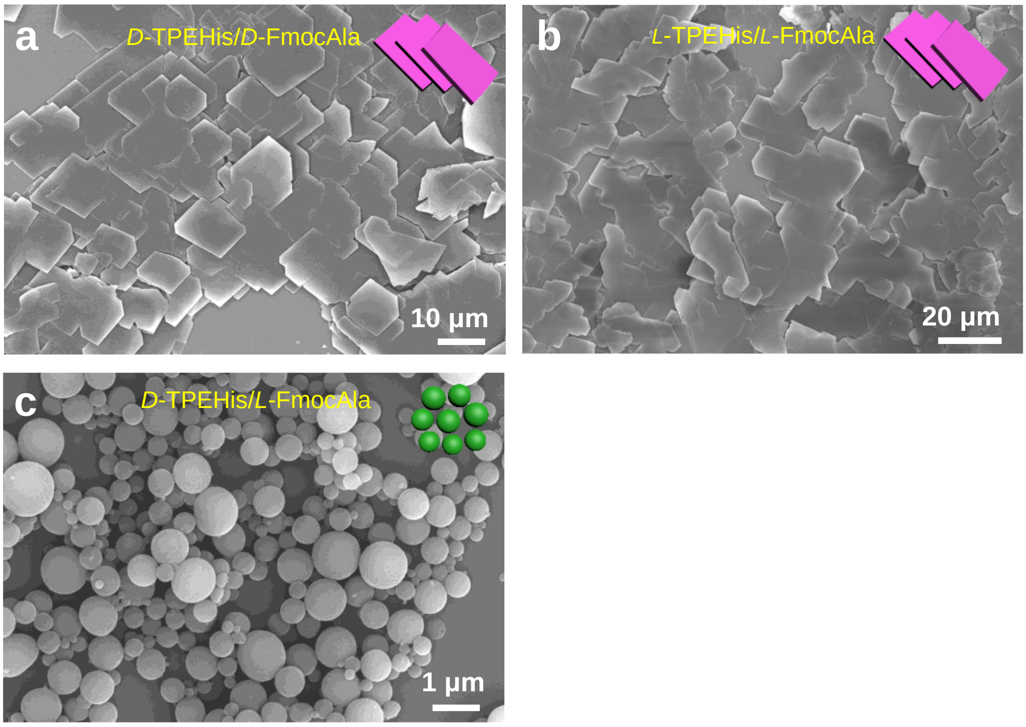

Supplementary Figure 17. SEM images of **a**. *D*-TPEHis/*D*-FmocAla, **b**. *L*-TPEHis/*L*-FmocAla and **c**. *D*-TPEHis/*L*-FmocAla co-assemblies.

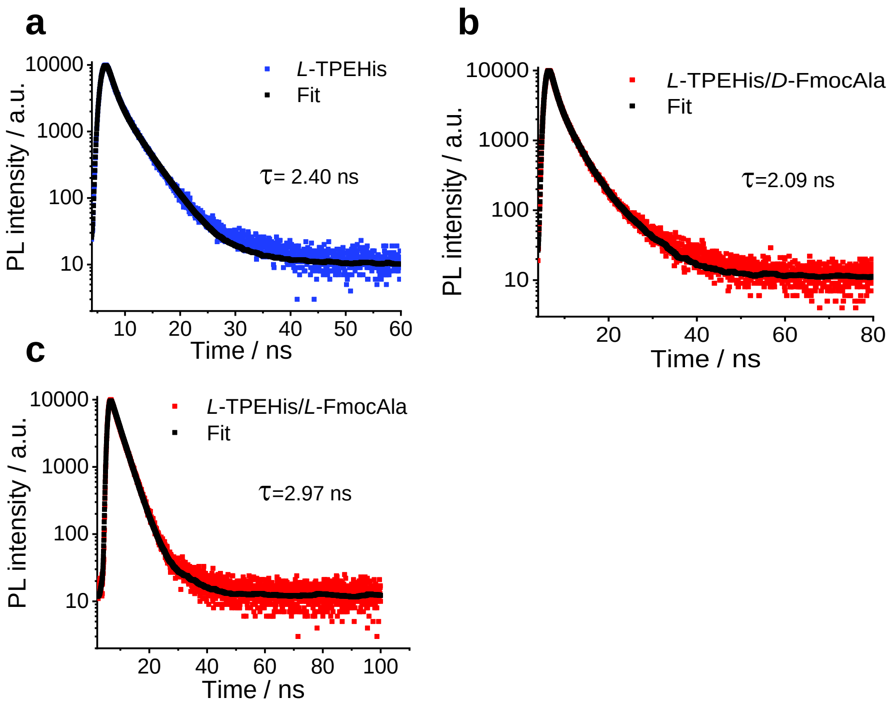

Supplementary Figure 18. Emission decay curves of **a**. *L*-TPEHis, **b**. *L*-TPEHis/*D*-FmocAla and **c**. *L*-TPEHis/*L*-FmocAla assemblies. Diode excitation wavelength is 320 nm, and monitor emission decay at 450 nm.

Supplementary Table 2. Summary of optical and chiroptical properties of *L*-TPEHis assemblies and *L*-TPEHis/*L*-FmocAla co-assemblies.

	λ_{abs} /nm	λ_{em} /nm	$\Delta\tilde{\nu}_{Stokes}$ /nm	FLQY[a]	Lifetime /ns	$\lvert g_{abs}\rvert$	$\lvert g_{lum}\rvert$	K_{fl} [10^8 S^{-1}][b]	K_{nr} [10^8 S^{-1}][c]
L-TPEHis	338	449	111	30%	2.40	3.0×10^{-3}	0.016	1.25	2.92
L-TPEHis/ *L*-FmocAla	338	450	112	76%	2.97	1.1×10^{-2}	0.14	2.56	0.81
L-TPEHis/ *D*-FmocAla	338	454	116	28%	2.09	5.2×10^{-5}	0	1.34	3.44

[a] Measured by absolute method using an integrating sphere, excitation wavelength 320 nm.
[b] $K_{fl} = \Phi_{fl}/\tau_{fl}$, where Φ_{fl} and τ_{fl} are the fluorescence quantum yield and lifetime, respectively.
[c] $K_{nr} = 1/\tau_{fl} - K_{fl}$.

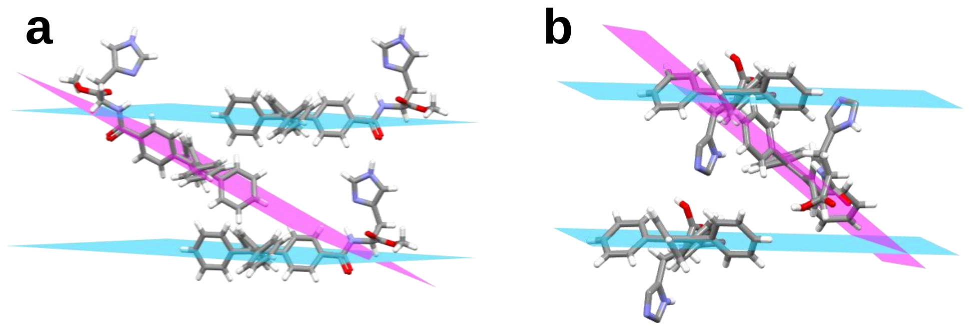

Supplementary Figure 19. The relative spatial orientation of three nearest TPE chromophores in the single crystal structure of **a**. *L*-TPEHis and **b**. *L*-TPEHis/*L*-FmocAla. The cyan and magenta planes are used to approximately represent the ethylene plane of TPE.

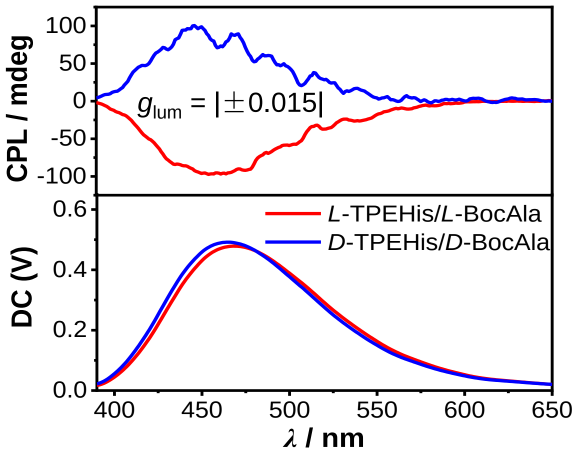

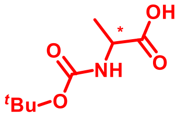

Boc-Ala

Supplementary Figure 20. CPL spectra of co-assemblies of *L*-TPEHis with *L*-BocAla and *D*- BocAla. [TPEHis] = [BocAla] = 10 mM in THF/H_2O, f_w = 90%, λ_{ex} = 320 nm.

6. Single crystal data

Supplementary Table 3. Single crystal data and structure refinement for *L*-TPEHis.

Identification code	*L*-TPEHis			
Empirical formula	C42H45N3O7			
Formula weight	703.81			
Temperature	170.00 K			
Crystal system	monoclinic			
Space group	P21			
Unit cell dimensions				
	a	9.6300(2) Å	α	90°
	b	9.3731(2) Å	β	95.524(2)°
	c	20.9366(5) Å	γ	90°
Volume	1881.02(7) $Å^3$			
Z	2			
Density (calculated)	1.243g/cm^3			
Absorption coefficient	0.686mm^{-1}			
F(000)	748.0			
Crystal size	0.03 × 0.02 × 0.01mm^3			
Radiation	CuKα (λ = 1.54184)			
Theta range for data collection	4.24 to 151.016°			
Index ranges	$-11 \le h \le 12$, $-11 \le k \le 11$, $-25 \le l \le 26$			
Reflections collected	24154			
Independent reflections	7419 [Rint = 0.0478, Rsigma = 0.0448]			
Data/restraints/parameters	7419/1/470			
Goodness-of-fit on F^2	1.036			
Final R indexes [I>=2σ (I)]	R1 = 0.0474, wR2 = 0.1208			
Final R indexes [all data]	R1 = 0.0603, wR2 = 0.1310			
Largest diff. peak and hole	0.35/-0.18 e $Å^{-3}$			

Supplementary Table 4. Single crystal data and structure refinement for *L*-TPEHis/*L*-FmocAla.

Identification code	*L*-TPEHis/*L*-FmocAla			
Empirical formula	C52.5H48N7O5.5			
Formula weight	864.98			
Temperature	170 K			
Crystal system	Orthorhombic			
Space group	P21212			
Unit cell dimensions				
	a	9.2965(5) Å	α	90°
	b	53.500(3) Å	β	90°
	c	9.4947(7) Å	γ	90°
Volume	4722.3(5) $Å^3$			
Z	4			
Density (calculated)	1.217 g/cm^3			
Absorption coefficient	0.647 mm^{-1}			
F(000)	1824.0			
Crystal size	0.02 × 0.01 × 0.005mm^3			
Radiation	CuKα (λ = 1.54184)			
Theta range for data collection	6.608 to 151.108°			
Index ranges	$-10 \leq h \leq 11$, $-66 \leq k \leq 65$, $-7 \leq l \leq 11$			
Reflections collected	28446			
Independent reflections	9192 [Rint = 0.1276, Rsigma = 0.1084]			
Data/restraints/parameters	9192/0/594			
Goodness-of-fit on F^2	1.174			
Final R indexes [I>=2σ (I)]	R1 = 0.1319, wR2 = 0.3255			
Final R indexes [all data]	R1 = 0.1940, wR2 = 0.3718			
Largest diff. peak and hole	1.03/-0.43e $Å^{-3}$			

7. Additional spectra

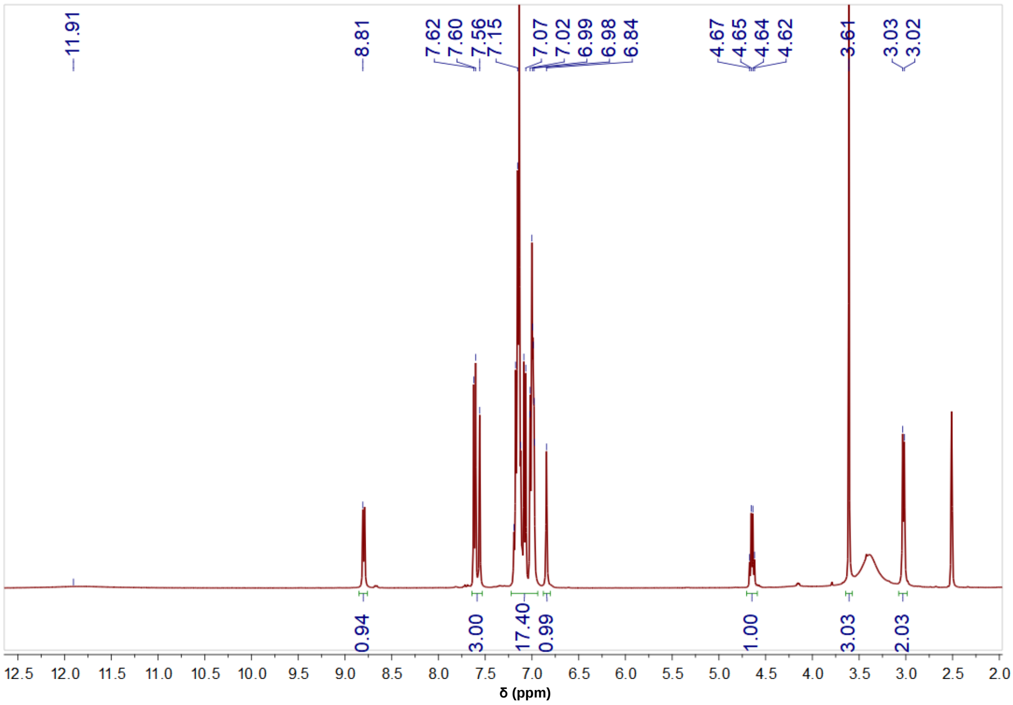

Supplementary Figure 21. ^{1}H-NMR spectrum of TPEHis (400 MHz, DMSO-d_6, 298K).

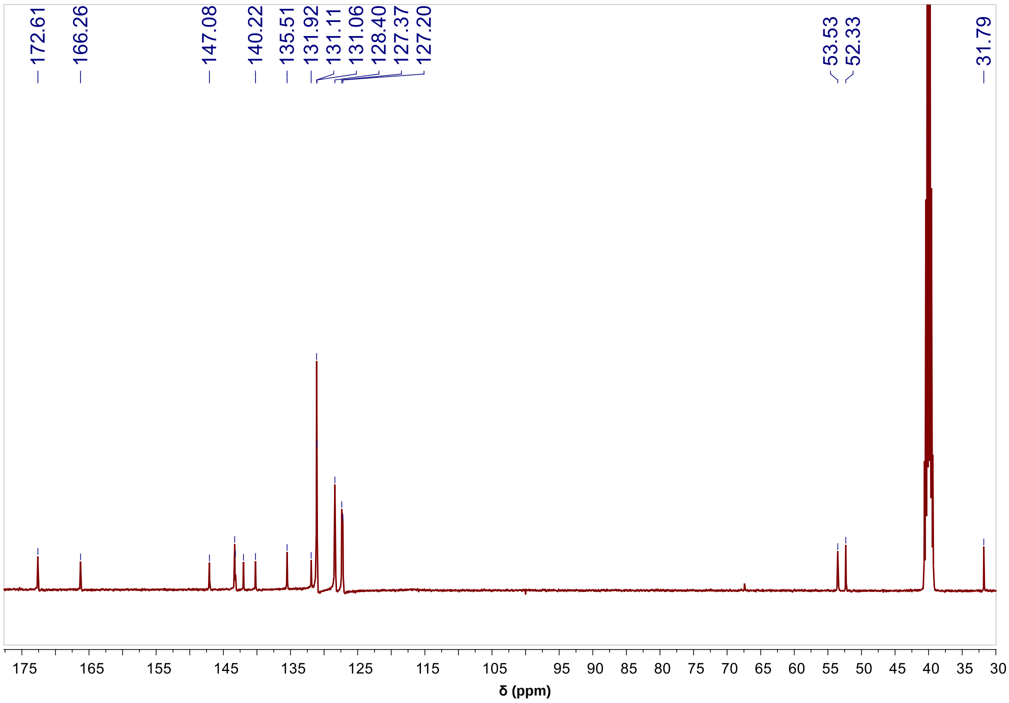

Supplementary Figure 22. ^{13}C-NMR spectrum of TPEHis (100 MHz, DMSO-d_6, 298K).

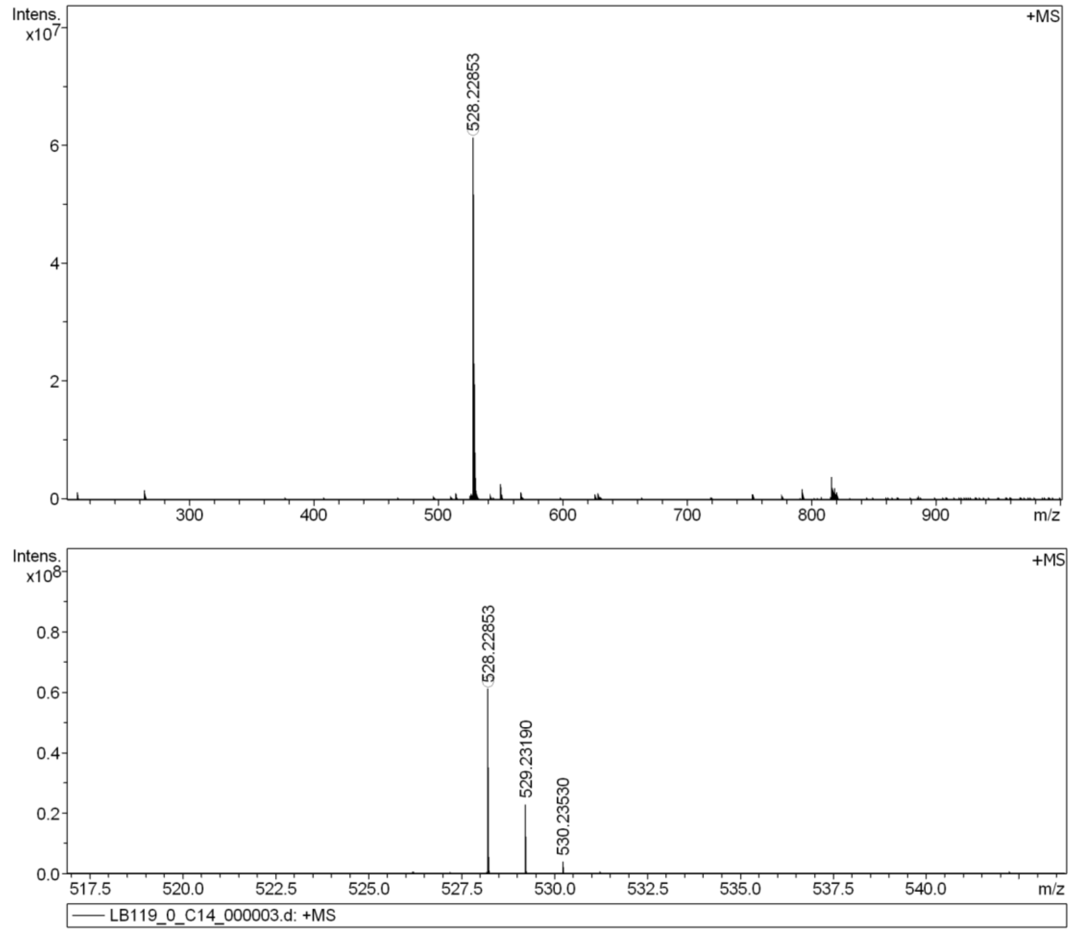

Meas. m/z	#	Ion Formula	Score	m/z	err [ppm]	Mean err [ppm]	mSigma	rdb	e^- Conf	N-Rule
528.228528	1	C34H30N3O3	100.00	528.228168	0.7	-0.8	4.5	21.5	even	ok

Supplementary Figure 23. HR-MALDI-FTICR mass spectrum of TPEHis (positive ion mode).

8. References

1. M. J. Frisch, G. W. Trucks, H. B. Schlegel, G. E. Scuseria, M. A. Robb, J. R. Cheeseman, G. Scalmani, V. Barone, B. Mennucci, G. A. Petersson, H. Nakatsuji, M. Caricato, X. Li, H. P. Hratchian, A. F. Izmaylov, J. Bloino, G. Zheng, J. L. Sonnenberg, M. Hada, M. Ehara, K. Toyota, R. Fukuda, J. Hasegawa, M. Ishida, T. Nakajima, Y. Honda, O. Kitao, H. Nakai, T. Vreven, J. A. Montgomery, Jr., J. E. Peralta, F. Ogliaro, M. Bearpark, J. J. Heyd, E. Brothers, K. N. Kudin, V. N. Staroverov, T. Keith, R. Kobayashi, J. Normand, K. Raghavachari, A. Rendell, J. C. Burant, S. S. Iyengar, J. Tomasi, M. Cossi, N. Rega, J. M. Millam, M. Klene, J. E. Knox, J. B. Cross, V. Bakken, C. Adamo, J. Jaramillo, R. Gomperts, R. E. Stratmann, O. Yazyev, A. J. Austin, R. Cammi, C. Pomelli, J. W. Ochterski, R. L. Martin, K. Morokuma, V. G. Zakrzewski, G. A. Voth, P. Salvador, J. J. Dannenberg, S. Dapprich, A. D. Daniels, O. Farkas, J. B. Foresman, J. V. Ortiz, J. Cioslowski, and D. J. Fox, Gaussian 09, Revision D.01, Gaussian, Inc., Wallingford CT, **2013**.

2. T. Lu, F. Chen, *J. Comput. Chem*. **2012**, *33*, 580-592.
